# Long-term mortality in young patients with spontaneous intracerebral haemorrhage: Predictors and causes of death

**DOI:** 10.1177/23969873211017723

**Published:** 2021-06-18

**Authors:** Jamie I Verhoeven, Marco Pasi, Barbara Casolla, Hilde Hénon, Frank-Erik de Leeuw, Didier Leys, Catharina JM Klijn, Charlotte Cordonnier

**Affiliations:** 1 Department of Neurology, Radboud University Medical Center, Donders Institute for Brain, Cognition and Behavior, Nijmegen, the Netherlands; 2 Inserm, CHU Lille, U1172-LilNCog-Lille Neuroscience and Cognition, University of Lille, Lille, France

**Keywords:** Intracerebral haemorrhage, young adults, long-term mortality

## Abstract

**Introduction:**

Intracerebral haemorrhage (ICH) in young adults is rare but has devastating consequences. We investigated long-term mortality rates, causes of death and predictors of long-term mortality in young spontaneous ICH survivors.

**Patients and methods:**

We included consecutive patients aged 18–55 years from the Prognosis of Intracerebral Haemorrhage cohort (PITCH), a prospective observational cohort of patients admitted to Lille University Hospital (2004–2009), who survived at least 30 days after spontaneous ICH. We studied long-term mortality with Kaplan-Meier analyses, collected causes of death, performed uni-/multivariable Cox-regression analyses for the association of baseline characteristics with long-term mortality.

**Results:**

Of 560 patients enrolled in the PITCH, 75 patients (75% men) met our inclusion criteria (median age 50 years, interquartile range [IQR] 44–53 years). During a median follow-up of 8.2 years (IQR 5.0–10.1), 26 patients died (35%), with a standardized mortality ratio of 13.0 (95% confidence interval [95% CI] 8.5–18.0) compared to peers from the general population. Causes of death were vascular in 7 (27%) patients, non-vascular in 13 (50%) and unknown in 6 (23%). Global cerebral atrophy (hazard ratio [HR] 3.0, 95% CI 1.1–8.6), modified Rankin Score >2 before ICH (HR 3.4, 95% CI 1.0–11.0), and excessive alcohol consumption (HR 3.3, 95% CI 1.1–10.2) were independently associated with long-term mortality.

**Discussion:**

We found a 13-fold higher mortality risk for young ICH survivors compared to the general French population. Predictors of long-term mortality were pre-existing conditions, not ICH-characteristics.

**Conclusion:**

Young ICH survivors remain at increased mortality risk of vascular and non-vascular death for years after ICH.

## Introduction

Spontaneous (non-traumatic) intracerebral haemorrhages (ICH) are rare in young adults, but are associated with poor functional outcomes and high mortality rates.^1^ The incidence ranges from 1.9 per 100.000 person-years in those 44 years and younger, to 19.0 per 100.000 persons-years between 45 and 55 years.^2^ ICH in young adults has devastating consequences, including a higher number of disability-adjusted life years and consequent increase of the socioeconomic burden.^3^ The main predictors of early mortality are similar to those found in older people, and are mainly related to ICH characteristics.^4,5^ However, causes and predictors of long-term mortality might differ from those of older people.^6,7^ Spontaneous ICH at a young age may indicate pre-existing brain fragility, possibly in combination with general poor health. This supposed brain fragility may be reflected by the presence of small vessel disease (SVD) markers on neuro-imaging. Currently, the predictive value for mortality of SVD markers, such as white matter hyperintensities, lacunes or cerebral microbleeds (CMBs), is unknown in young survivors of spontaneous ICH.

The aim of this study was to investigate the long-term mortality rate, causes of death and predictors of long-term mortality in young 30-day survivors of ICH.

## Methods

### Study design and patient selection

We included patients from the Prognosis of Intracerebral Haemorrhage Cohort (PITCH),^8^ a prospective observational cohort of consecutive patients with ICH (first-ever and recurrent), confirmed on imaging, admitted to Lille University Hospital between November 2004 and April 2009.^8^ Exclusion criteria were: pure intraventricular haemorrhage, ICH resulting from vascular malformation, cerebral venous thrombosis, head trauma, intracranial tumour, haemorrhagic transformation of an ischaemic stroke and patients referred from other hospitals.^8,9^ For this study we included adults aged from 18 to 55 years at onset, who were still alive at 30 days following index event. The design of the PITCH cohort was in line with the PROGRESS (Prognosis Research Strategy) recommendations.^8,10^

### Baseline clinical data

We collected age, sex and level of education (dichotomized in less or more than eight years of education). We recorded history of previous ischaemic stroke, ICH, transient ischaemic attack (TIA), ischaemic heart disease, arterial hypertension, diabetes mellitus and atrial fibrillation, as previously reported.^8^ We defined current smoking as everyday smoking or cessation less than 1 year before, excessive alcohol consumption as a weekly consumption of more than 300 grams of alcohol (30 units),^8,11^ and hypercholesterolemia as a total cholesterol level of 2.3 g/l or more, or any treatment by statin or fibrate (except when given for coronary reasons).^8^ We also collected the use of antihypertensive and antidiabetic drugs, statins, antiplatelet and anticoagulation drugs at admission, as previously stated.^8,11^ The pre-existing level of dependency was assessed with the modified Rankin Scale (mRS), with a pre-ICH score of 0–2 indicating independency and mRS pre-ICH >2 indicating dependency.^12^ We evaluated the severity of the neurological deficit at admission with the National Institutes of Health Stroke Scale (NIHSS).^13^

### Baseline radiological assessment

At admission all patients underwent a brain CT-scan. The location of the ICH was classified into deep (lenticular or caudate nuclei, internal or external capsule or posterior fossa if the haemorrhage originated from the brainstem or cerebellum) or lobar (frontal, temporal, parietal or occipital origin of the haemorrhage), according to the origin of bleeding.^11^ There were six patients with multiple localizations of ICH, all locations were scored separately in the different location categories. We recorded intraventricular breakthrough and the presence of blood in the fourth ventricle. The volume of the haemorrhage was measured according to the AxBxC/2 method.^9^

During hospitalization all patients underwent at least one CT-angiogram or MR-angiogram of the brain to rule out structural underlying causes of the ICH.^8^

All patients without contra-indications underwent a 1.5 Tesla Brain MRI scan, including at least fluid-attenuated inversion recovery (FLAIR), and T2*gradient-echo (GRE) weighted sequences (echo time: 22·8 ms, repetition time: 700 ms; flip angle: 25°; field of view: 250 mm; matrix: 352 × 224; slice thickness: 5 mm; and interslice gap: 1·5 mm).^14^

For every MRI, we evaluated the presence of white matter hyperintensities using the Fazekas 4-point score. The presence of lacunes was evaluated following the definition of a lacune being deep, subcortical or pontine ovoid lesions (3–15mm) with cerebrospinal fluid-like signal with or without hyperintense fluid-attenuated inversion recovery border.^15^ Cerebral microbleeds were defined as round foci of hypointense signal (≤10 mm) in the brain parenchyma of T2* Gradient-Echo-Weighted images and rated using the Brain Observer Microbleed Scale (BOMB).^15^ We assessed global cortical atrophy according to the 4-point scale (Global Cortical Atrophy Scale)^16^. Finally, we calculated a composite score of small vessel disease burden according to the simple SVD score.^17^

### Follow-up

All patients were followed up at 6 months, 12 months and annually thereafter.^18^ For this study, we retrospectively obtained the causes of death through medical records, contact with general practitioners (GP) or other physicians listed in the medical records. We classified the primary causes of death into four categories, according to a previously reported classification:^19^ vascular deaths (recurrent ICH, ischaemic stroke, other), non-vascular deaths and death from an unknown cause due to lack of information. Non-vascular deaths were divided into infection-related (death presumed as a direct result of the infection or due to systemic complications or organ failure reasonably linked to the infection), cancer or malignant hemopathies (death presumed as a direct result of an identified malignancy or metastases, or death due to secondary complications reasonably linked to the underlying malignancy) and metabolic causes (death presumed as a direct result of or complications of a metabolic disorder, such as death resulting from liver cirrhosis due to hepatic encephalopathy or portal hypertension).

### Statistical analysis

We expressed quantitative variables as mean with standard deviation (SD), or as a median values with interquartile range (IQR), as appropriate. We expressed categorical variables as a number with percentages. We assessed the risk of all-cause long-term mortality through Kaplan-Meier analyses. We calculated the number of person-years of each individual patient from the date of ICH to the date of death or the date of final follow-up. We calculated cumulative mortality at 1, 5 and 10 years with 95% confidence intervals following the index ICH. Additionally, we calculated the annual risk of mortality with the following formula: 1-([1-Ic]^[1/n]^), where Ic is the cumulative mortality at n (number) of years after index event.

We calculated standardized mortality ratios (SMRs) by dividing the observed deaths in our cohort with the number of expected deaths or peers from the general French population, matched by age, sex and calendar year. The expected mortality rates were retrieved from the global Human Mortality Database (http://www.mortality.org).^20^

We used dichotomized variables for the MRI markers. Global cerebral atrophy was scored as absent (0–1) and present (2–3), white matter hyperintensities as absent (0–1) or present (2–3), and lacunes and cerebral microbleeds as absent or present.

We assessed the association of baseline characteristics with long-term mortality through Cox Proportional Hazard models, expressed through hazard ratios with 95% confidence intervals and corresponding p-values. First, we tested the association of all baseline characteristics with long-term mortality through univariate cox-regression analyses. We subsequently added all characteristics with a univariate p-value of <0.10 simultaneously into a multivariable cox proportional hazards model. The area under the curve of the multivariable prediction model was calculated through Harrell’s C-statistic. Assumption of proportionality in the cox-regression model was evaluated and confirmed through calculating Schoenfeld residuals. Finally, we performed descriptive analyses for the different causes of death and calculated mean time to death for each category.

Statistical significance was set at 0.05 with two-sided p-values for all analyses.

We performed statistical analyses with Excel (Microsoft Office version 16.28), SPSS (IBM version 26) and R Project for Statistical Computing.

### Ethics protocol approval and patients consent

The study protocol was regarded as observational by the Internal Review Board of the Lille University Hospital, which granted ethics approval for this study. Patients, or their relatives or primary caregiver, gave informed consent for follow-up.

### Data availability

The raw and anonymized data used in this study can be shared upon request from a qualified investigator and authorization of our IRB.

## Results

### Patient inclusion

**Figure 1.**
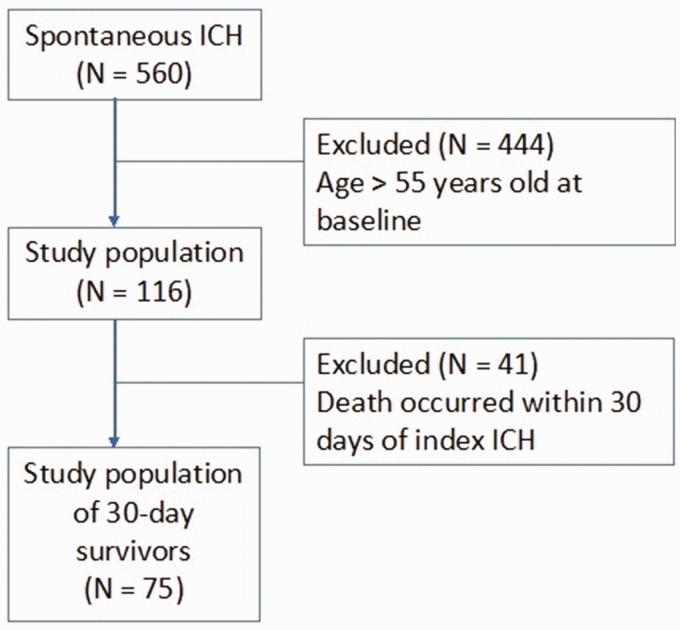
Flowchart of patient inclusion. ICH = intracerebral hemorrhage, N = number.

Of the 560 patients with spontaneous ICH included in the PITCH Cohort, 116 (20.7%) were between 18 and 55 years at the time of ICH. Forty-one patients (35.3%) were excluded because they died within 30 days of their index event. This resulted in a cohort of 75 patients (Figure 1).

### Baseline clinical and radiological characteristics

Of the 75 patients included in this study, 56 (74.7%) were men. The median age was 50 (IQR 44–53) years. Table 1 summarizes clinical and radiological baseline characteristics. MRI-scans were performed in 64 patients (85.3%), with a median ICH-MRI interval of 6 (IQR 3–9.8) days.

**Table 1.** Baseline clinical and radiological characteristics of 30-day survivors of ICH in patients 18–55 years.

Demographics		Neurological status at baseline	
Total	75 (100)	Mean NIHSS (SD)	11.5 (9.2)
Mean age, years (SD)	47.8 (7.2)	CT characteristics	
Male sex	56 (74.7)	Location^‡^	
≥8 years of education^†^	39 (54.9)	Brainstem	13 (17.3)
mRS pre-ICH > 2	9 (12.0)	Cerebellar	2 (2.7)
Medical history		Deep	42 (56.0)
Arterial hypertension	37 (49.3)	Lobar	23 (30.7)
Diabetes mellitus	9 (12.0)	Undetermined	1 (1.3)
Hypercholesterolemia	13 (17.3)	Intraventricular haemorrhage	22 (29.3)
Smoking	34 (45.3)	ICH-volume (mL); mean (SD)^§^	18.3 (21.7)
Excessive alcohol consumption	31 (41.3)	MRI features	
Previous ICH	7 (9.3)	MRI performed during admission	64 (85.3)
Previous ischaemic stroke	5 (6.7)	White matter hyperintensities	19 (29.7)
Previous TIA	2 (2.7)	≥1 Lacunes	22 (34.4)
Previous myocardial infarction	4 (5.3)	≥1 Microbleeds	27 (42.2)
Treatment used at baseline		Global cortical atrophy	11 (17.2)
Antihypertensive therapy	29 (38.7)	Simple SVD score^∥^	
Statins	10 (13.3)	Simple SVD score 0	32 (50.0)
Antiplatelet therapy	13 (17.3)	Simple SVD score 1	16 (25.0)
Oral anticoagulation therapy	3 (4.0)	Simple SVD score 2	9 (14.1)
		Simple SVD score 3	7 (10.9)

Data listed are numbers (%) or median (IQR), unless otherwise specified. † Level of education known in 71 patients, % out of known number of patients. ^‡^Six cases with multiple ICH locations, therefore this adds up to 81 ICH-locations. ^§^ICH volume calculated with the AxBxC/2 method. ^∥^Simple SVD score calculated according to Staals et al.^26^ Abbreviations: ICH = intracerebral haemorrhage, mRS = modified Rankin Score, TIA = transient ischaemic attack.

### All-cause long-term mortality

**Figure 2.**
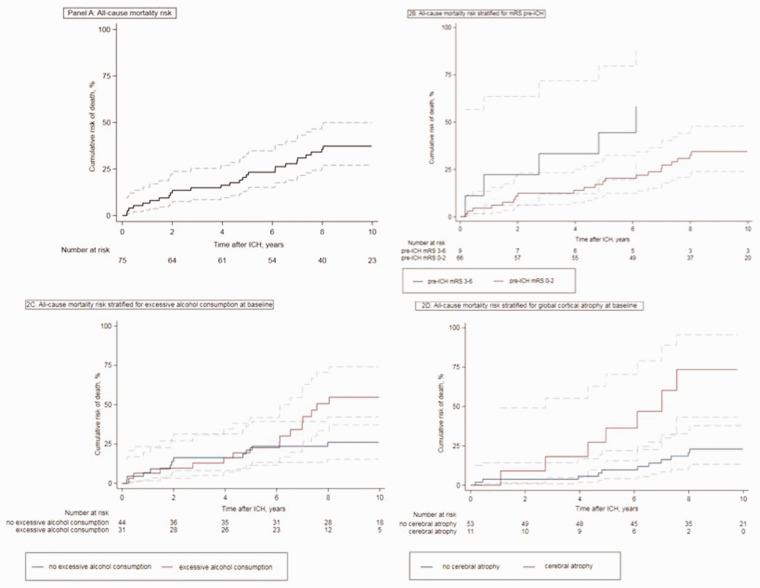
All-cause cumulative mortality risk. (a) All-cause mortality risk. (b) All-cause mortality risk stratified for mRS pre-ICH. (c) All-cause mortality risk stratified for excessive alcohol consumption at baseline. (d) Panel D: all-cause mortality risk stratified for global cortical atrophy at baseline.Dotted lines represent 95% confidence intervals. P-values according to log-rank analysis.

During a median follow-up period of 8.2 (IQR 5.0–10.1) years, 26 patients died (34.7%). In the first year following the index ICH 5 patients (6.7%, 95% CI 2.8–15.3) had died and after 5 years 16 patients (21.7%, 95% CI 13.9–33.0) had died. After 10 years, the cumulative mortality was 37.2% (95% CI 27.0–49.8) (Figure 2, panel (a)). The mean annual mortality risks remained stable over time.

The standardized mortality ratio (SMR) was 13.0 (95% CI 8.5–18.0, p < 0.001) for the young ICH patients compared to matched peers from the general population. The SMR was 15.7 (95% CI 9.4–22.7, p < 0.001) for men and 8.3 (95% CI 2.8–15.2, p < 0.001) for women.

### Causes of long-term mortality

The cause of death was identified in 20 out of 26 patients (76.9%). Seven patients died from a vascular cause (35%) after a median period of 1.1 (inter quartile range [IQR] 0.3–4.8) years. Three patients died of long-term complications of the index ICH. Four patients died from a new stroke, a recurrent ICH in two and acute neurological worsening attributed to a new stroke of undetermined type in two. Thirteen patients died from a non-vascular cause (65%) after a median period of 5.0 (IQR 2.1–7.2) years (Table 2). Three were due to sepsis, three to metabolic disorders, in all cases the result of liver failure due to excessive alcohol consumption and seven were due to malignancy. Five patients died from lung cancer and two from hepatic carcinomas. All were smokers and six out of seven had a history of excessive alcohol consumption. The cancer had been diagnosed before ICH in one patient, diagnosed in the acute stage of the ICH in one patient, and diagnosed during follow-up in the other five.

**Table 2.** Causes of death in 30-day survivors of ICH aged 18 to 55 years old.

	N (%)	Median time to death, years (IQR)
Total	26 (100)	4.5 (1.4–6.6)
*Vascular death*	7 (26.9)	1.1 (0.3–4.8)
Neurovascular	7 (26.9)	
Death due to complications of index stroke	3 (11.5)	
Death due to a new stroke	4 (15.4)	
Other vascular	0 (0.0)	
Coronary death	0 (0.0)	
Other vascular event (e.g. pulmonary embolism)	0 (0.0)	
*Non-vascular death*	13 (50.0)	5.0 (2.1–7.2)
Infection-related death	3 (11.5)	
Cancer or malignant hemopathies	7 (26.9)	
Metabolic disorder	3 (11.5)	
Unknown	6 (23.1)	4.9 (2.0–6.5)

Abbreviations used: IQR = interquartile range.

### Baseline clinical and radiological characteristics associated with long-term mortality

In univariable analysis global atrophy on MRI was the only significant predictor of long-term mortality with a hazard ratio of 4.7 (95% CI 1.8–12.5) (Table 3).

**Table 3.** Univariable analyses of possible predictors of long-term mortality in young 30-day survivors of ICH.

Demographics	Patients who died at end of follow up	Survivors at end of follow up	p-value	Hazard ratio(95% CI)
Total	N = 26	N = 49		
Mean age, years (SD)	49.9 (4.5)	46.7 (8.03)	0.105	1.1 (1.0–1.2)
Male sex	20 (76.9)	36 (73.5)	0.812	1.2 (0.5–2.8)
≥8 Years of education^†^	10 (38.5)	29 (59.2)	0.126	0.5 (0.2–1.2)
mRS pre-ICH >2	5 (19.2)	4 (8.16)	*0.087*	*2.4 (0.9*–*6.3)*
Medical history				
Arterial hypertension	15 (57.7)	22 (44.9)	0.361	1.4 (0.7–3.1)
Diabetes mellitus	4 (15.4)	5 (10.2)	0.496	1.5 (0.5–4.2)
Hypercholesterolemia	4 (15.4)	9 (18.4)	0.839	0.9 (0.3–2.6)
Smoking	15 (57.7)	19 (38.8)	0.119	1.9 (0.9–4.1)
Excessive alcohol consumption	15 (57.7)	16 (32.7)	*0.061*	*2.1 (1.0*–*4.6)*
Previous ICH	4 (15.4)	3 (6.1)	0.180	2.1 (0.7–6.0)
Previous ischaemic stroke	2 (7.7)	3 (6.1)	0.851	1.2 (0.3–4.9)
Previous myocardial infarction	2 (7.7)	2 (4.1)	0.351	2.0 (0.5–8.4)
Treatment used at baseline				
Antihypertensive therapy	12 (46.2)	17 (34.7)	0.323	1.5 (0.7–3.2)
Statins	2 (7.7)	8 (16.3)	0.370	0.5 (0.1–2.2)
Antithrombotic therapy	7 (26.9)	9 (18.4)	0.403	1.5 (0.6–3.5)
Neurological status at presentation				
Mean NIHSS (SD)	10.0 (9.7)	12.2 (8.9)	0.253	1.0 (0.9–1.0)
ICH characteristics at CT				
Deep location	15 (57.7)	37 (75.5)	0.136	0.6 (0.3–1.2)
Intraventricular haemorrhage	8 (30.8)	14 (28.6)	0.776	1.1 (0.5–2.6)
Mean ICH-volume^‡^ (SD)	16.9 (24.7)	19.0 (20.2)	0.594	1.0 (1.0–1.0)
MRI features				
White matter hyperintensities	7 (38.9)	12 (26.1)	0.280	1.7 (0.7–4.4)
≥1 Lacunes	8 (44.4)	14 (30.4)	0.177	1.9 (0.8–4.9)
≥1 Microbleeds	7 (38.9)	20 (43.5)	0.172	1.8 (0.8–4.4)
Global cortical atrophy	7 (38.9)	4 (8.7)	*0.002*	*4.7 (1.8*–*12.5)*
Simple SVD score ∥ ≥ 1	9 (34.6)	23 (46.9)	0.96	1.0 (0.4–2.6)
Simple SVD score ∥ ≥ 2	7 (34.6)	9 (22.4)	0.168	2.0 (0.8–6.0)
Simple SVD score ∥ ≥ 3	3 (11.5)	4 (8.2)	0.538	1.5 (0.4–5.1)

Data listed are numbers (%), unless otherwise specified. ^†^Level of education known in 71 patients, % out of known number of patients. ^‡^ ICH volume calculated with the AxBxC/2 method. ∥ simple SVD score calculated according to Staals et al. and hazard ratio’s compared to Simple SVD score of 0.^26^Values with a p-value of <0.10 are italicized.

In multivariable analysis, including pre-ICH dependency (HR 3.4, 95% CI 1.0–11.0), excessive alcohol consumption (HR 3.3, 95% CI 1.1–10.2), and global cerebral atrophy (HR 3.0, 95% CI 1.1–8.6) in the model, all three were independently associated with long-term mortality (Table 4 and Figure 2 panels (b) to (d)). The fit of this model was good, with an area under the curve of 0.7 (Harrell’s C). The separate SVD markers or SVD score were not associated with long-term mortality.

**Table 4.** Multivariable analysis of predictors associated with long-term mortality in 30-day survivors of ICH.

	Hazard ratio (95% CI)	p-value according to multivariable cox-regression
Clinical and radiological predictors^†^		
mRS pre-ICH >2	3.4 (1.0–11.0)	**0.044**
Excessive alcohol consumption	3.3 (1.1–10.2)	**0.039**
Presence of global cortical atrophy	3.0 (1.1–8.6)	**0.039**

^†^Predictors were chosen for the multivariable model if p < 0.1 in the univariate cox-regression analyses (Table 2).Values with p-value <0.05 are in bold.

## Discussion

In a prospective cohort of 75 consecutive young 30-day survivors of ICH, we found a substantial risk of long-term mortality of around 37%, which is 13 times higher compared to peers from the general population. Causes of death were vascular disease in one out of four patients and non-vascular causes, including infections, malignancies, and metabolic disorders in half of patients. Independent predictors of death were excessive alcohol consumption at baseline, pre-stroke dependency and global cortical atrophy on MRI.

The long-term risk of mortality in young 30-day survivors of ICH in our study was higher than in two previously published hospital-based prospective cohort studies. One Finnish study found a cumulative mortality of 9.5% in a median follow-up of 9.7 years and a Dutch study found a cumulative mortality of 12.8% during a mean follow-up of 11.3 years.^6,7^ It is also higher than in one Dutch population-based study, that demonstrated a 10-year cumulative mortality of 15.7% in young ICH survivors and a SMR of 8.4 (95% CI 7.4–9.3).^21^ This could be explained in several ways. First, our inclusion-criteria differed from the previous studies in upper age-limit and causes of ICH. We used an upper age limit of 55 years instead of 49 in the three previously mentioned studies. Higher age was positively associated with a higher risk of mortality in the Dutch population-based and hospital-based study.^6,21^ However, age was not a significant predictor of the risk of long-term mortality in the Finnish study, nor in our own study.^7^ Second, in our study all patients with secondary causes of ICH, such as vascular malformations, were excluded. Whereas in the previous three studies, ICH due to secondary causes were included. Third, the patients in our study may have had a higher background mortality-risk, independent of the ICH. This study was carried out in the north of France, where the proportion of smokers and excessive alcohol consumption is high compared to other European places.^11^ The proportion of excessive alcohol users in our cohort was higher than the Dutch hospital-based cohort.^6^ The Finnish study and Dutch population-based study did not report smoking or alcohol use.^7,21^

This study is the first to include functional status before ICH and MRI-characteristics of SVD as predictors of long-term mortality in young ICH survivors. These factors might be relevant for predicting long-term mortality because they reflect early brain damage due to vascular risk factor burden and underlying health conditions. Both pre-stroke dependency and cerebral global atrophy were found to be predictors of long-term mortality in the PITCH cohort when including 30-day survivors of all ages, as well as higher age, male sex, higher NIHSS at baseline and any recurrent stroke or dementia during follow-up.^18^

In these young 30-day ICH survivors, cerebral atrophy was the only radiological predictor of long-term mortality. An explanation could be that cerebral atrophy can be considered as an expression of severe brain damage secondary to both cerebral small vessel disease and neurodegeneration (e.g. Alzheimer disease type) or associated to excessive alcohol consumption.

We have also shown that pre-stroke dependency and excessive alcohol consumption at baseline are independently associated with a higher long-term mortality risk. Excessive alcohol consumption was not found to be predictive of long-term mortality in the PITCH cohort of all ages and therefore may be specific to this young subgroup of this cohort.^18^ Previously, excessive alcohol consumption has been shown to be associated with a higher mortality risk in ICH patients of all ages.^11,22^

The Finnish hospital-based study found male sex and diabetes at baseline to be predictors of long-term mortality, however they did not take alcohol use, mRS pre-ICH or MRI-markers into account.^7^ The Dutch population-based study found age, male sex, a higher number of comorbidities (Charlson Comorbidity index) and longer hospital stay to be associated with an increased risk of long-term mortality.^7,21,23^ The Charlson Comorbidity index includes various diseases such as liver disease, dementia and cardiovascular disorders. These may reflect liver failure due to excessive alcohol consumption and cerebral damage or atrophy due to cardiovascular burden or neurodegenerative conditions. Therefore, the predictive value of higher comorbidity found in the Dutch population-based study may mirror the predictive value of excessive alcohol consumption, mRS pre-ICH >2 and global cerebral atrophy found in our study.

Our finding that non-vascular causes were the main cause of death in our population of young ICH patients, supports the hypothesis that the increased long-term mortality rate is driven by underlying risk-factors and/or health conditions and not factors directly related to the ICH itself. The high number of malignancies and alcohol-related deaths may be explained by the high percentage of shared risk-factors such as smoking for ICH as well as cancer, and excessive alcohol consumption for ICH as well as metabolic deaths. Additionally, cancer influences the coagulation system and therefore may play a role in causing the ICH, and therefore ICH may even be a first symptom of an underlying malignancy.^24,25^ In the Dutch hospital-based cohort, the percentage of vascular deaths was higher reaching 70%, whereas in the population-based cohort the percentage of vascular deaths was similar to our cohort with 29.8%.^6,21^ Cause of death was not reported in the Finnish study.^7^

Strengths of our study are the prospective design with systematic and detailed data collection, the long follow-up period, and the fact we were able to study small vessel disease on MRI in most patients. However, this study also has some limitations. First, although the total number of patients was large for this specific subgroup, the absolute number was still limited. A second limitation is that we did not have any information on illicit drug use in our study population, which is a relevant risk-factor for ICH at a young age.^27^ Third, MRI was not performed in all patients and therefore there is a risk of underestimating the association between MRI-markers and mortality risk. Fourth, we acknowledge a partial overlap between this cohort and the one of a recent published study.^18^ However, the target population and research question are different.

## Conclusions

The high long-term mortality we found in these young ICH survivors is worrisome and needs to be clarified further in prospective studies with larger patient numbers that combine extensive clinical and MRI phenotyping with a long follow-up period. Our results demonstrate that vascular risk factors such as excessive alcohol use are a serious issue in this population and need to be addressed. Additionally, our results show that these young ICH survivors remain at increased mortality risk of vascular and non-vascular death years after their ICH and therefore long-term multidisciplinary follow-up is needed.
